# Supplementary material for: Prostaglandin E2 receptor Ptger4b regulates female-specific peptidergic neurons and female sexual receptivity in medaka
Source: Commun Biol. 2022 Nov 10;5:1215. doi: 10.1038/s42003-022-04195-x (PMC9649691; doi:10.1038/s42003-022-04195-x)
Supplement: Supplementary file 2 — Supplementary Information [file 42003_2022_4195_MOESM2_ESM.pdf]

**Supplementary Table 1. Species names and GenBank accession numbers of the protein sequences used in this study.**

| protein | species | accession number |
|---|---|---|
| CARTPT | human (*Homo sapiens*) | NP_004282 |
| CARTPT | mouse (*Mus musculus*) | NP_038760 |
| Cartpt | *Xenopus laevis* | NP_001087565 |
| Cartpt1 | zebrafish (*Danio rerio*) | ADB12484 |
| Cartpt2a | zebrafish (*Danio rerio*) | ADB12485 |
| Cartpt2b | zebrafish (*Danio rerio*) | ADB12486 |
| Cartpt2b | medaka (*Oryzias latipes*) | NP_001191710 |
| Cartpt3 | zebrafish (*Danio rerio*) | ADB12487 |
| Cartpt (ch3) | medaka (*Oryzias latipes*) | NP_001191708 |
| Cartpt (ch4) | medaka (*Oryzias latipes*) | NP_001191709 |
| Cartpt (ch9) | medaka (*Oryzias latipes*) | NP_001191724 |
| Cartpt (ch11) | medaka (*Oryzias latipes*) | NP_001191711 |
| Cartpt (ch22) | medaka (*Oryzias latipes*) | NP_001191712 |
| TAC1 | human (*Homo sapiens*) | NP_003173 |
| TAC1 | mouse (*Mus musculus*) | NP_033337 |
| Tac1 | *Xenopus tropicalis* | NP_001165757 |
| Tac1 | zebrafish (*Danio rerio*) | NP_001243320 |
| Tac1 | medaka (*Oryzias latipes*) | BAH03329 |
| TAC3 | human (*Homo sapiens*) | NP_037383 |
| TAC3 | mouse (*Mus musculus*) | NP_033338 |
| Tac3 | *Xenopus tropicalis* | NP_001254820 |
| Tac3 | medaka (*Oryzias latipes*) | NP_001265832 |
| Tac3a | zebrafish (*Danio rerio*) | NP_001243318 |
| Tac3b | zebrafish (*Danio rerio*) | NP_001243319 |
| TAC4 | human (*Homo sapiens*) | NP_733786 |
| TAC4 | mouse (*Mus musculus*) | NP_444323 |
| Tac4a | zebrafish (*Danio rerio*) | NP_001256965 |
| Tac4a | medaka (*Oryzias latipes*) | XP_020561189 |
| Tac4b | zebrafish (*Danio rerio*) | XP_017213989 |
| Tachykinin | *Ciona intestinalis* | NP_001027966 |
| Tachykinin | *Drosophila melanogaster* | NP_650141 |
| PTGER4 | human (*Homo sapiens*) | NP_000949 |
| PTGER4 | mouse (*Mus musculus*) | NP_001129551 |
| Ptger4 | chicken (*Gallus gallus*) | NP_001074972 |
| Ptger4 | *Xenopus tropicalis* | NP_001120554 |
| Ptger4 | medaka (*Oryzias latipes*) | XP_020561449 |
| Ptger4 | medaka (*Oryzias latipes*) | XP_020566514 |
| Ptger4a | zebrafish (*Danio rerio*) | NP_001034718 |
| Ptger4b | zebrafish (*Danio rerio*) | NP_001121839 |

| | | |
|---|---|---|
| Ptger4b | medaka (*Oryzias latipes*) | NP_001295903 |
| Ptger4c | zebrafish (*Danio rerio*) | NP_001268925 |
| PTGER2 | human (*Homo sapiens*) | NP_000947 |
| PTGER3 | human (*Homo sapiens*) | NP_942011 |

**Supplementary Table 2. Primers used in this study.**

| target | direction | purpose | sequence (5′ to 3′) |
|---|---|---|---|
| *ptger4b* | forward | real-time PCR | GGTTCTCATCTGCTCCATACCTTT |
| *ptger4b* | reverse | real-time PCR | GGGGTTAATGGAAGCCATGC |
| *actb* | forward | real-time PCR | CCCCACCCAAAGTTTAG |
| *actb* | reverse | real-time PCR | CAACGATGGAGGGAAAGACA |
| *eef1a* | forward | real-time PCR | AGAAGGAAGCCGCTGAGATG |
| *eef1a* | reverse | real-time PCR | AGAGCGATGTCGATGGTGATAC |
| *ptger4b* | forward | genotyping (gDNA PCR) | ACCATCGCCATCTACATGAAG |
| *ptger4b* | reverse | genotyping (gDNA PCR) | GATGAAGCGTTTGTGCATGAG |
| *ptger4b* | forward | genotyping (CS) | CATCTACATGAAGGGCTCCTG |
| *ptger4b* | forward | genotyping (HRM) | GCATCTACATGTTCAACGCCTTT |
| *ptger4b* | reverse | genotyping (HRM) | TGTTCCGCCAGTCGATGAAG |

gDNA PCR, PCR on genomic DNA; CS, cycle sequence; HRM, high resolution melt analysis.

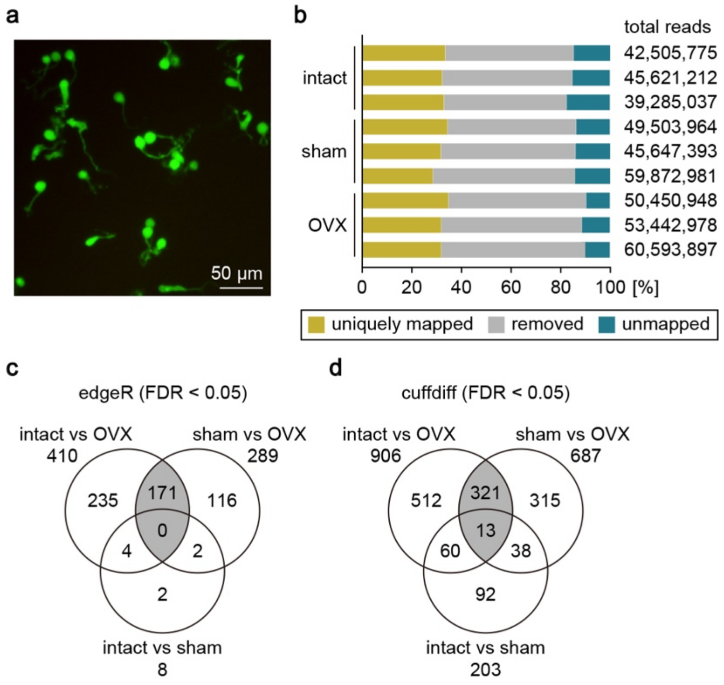


**Supplementary Fig. 1 RNA-seq of female-specific preoptic neurons.** **a** Representative micrograph of GFP-labeled *npba*-expressing neurons isolated from the PMm/PMg. Scale bar represents 50 µm. **b** Summary of total reads and percentages of uniquely mapped reads (mapped reads after removing multi-mapped and duplicate reads) removed reads (multi-mapped and duplicate reads), and unmapped reads for each sample (n = 3 for each of intact, sham-operated (sham), and ovariectomized (OVX) females). Venn diagrams showing the number of genes that are differentially expressed (false discovery rate (FDR) < 0.05) in intact, sham, and OVX females as determined by edgeR (**c**) and cuffdiff (**d**).

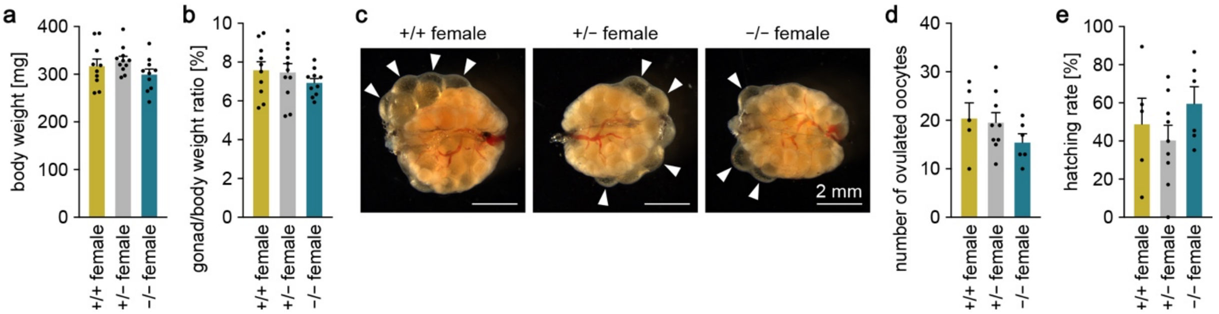


**Supplementary Fig. 2 *ptger4b*-deficient females retain normal ovarian development and function—Δ10 line.** Body weights (**a**) and gonad/body weight ratios (**b**) of adult *ptger4b*$^{+/+}$ (n = 10), *ptger4b*$^{+/−}$ (n = 11), and *ptger4b*$^{−/−}$ (n = 10) females of the Δ10 line. **c** Representative images of the ovaries of adult *ptger4b*$^{+/+}$, *ptger4b*$^{+/−}$, and *ptger4b*$^{−/−}$ females of the Δ10 line. Arrowheads indicate representative ovulated oocytes. Scale bars represent 2 mm. Number of ovulated oocytes (**d**) and hatching rate of fertilized eggs (**e**) of *ptger4b*$^{+/+}$ (n = 5), *ptger4b*$^{+/−}$ (n = 9), and *ptger4b*$^{−/−}$ (n = 6) females of the Δ10 line. Quantitative data were expressed as means with error bars representing standard error of the mean. Statistical differences were assessed by Bonferroni's *post hoc* test (**a**, **b**, **d**, **e**).

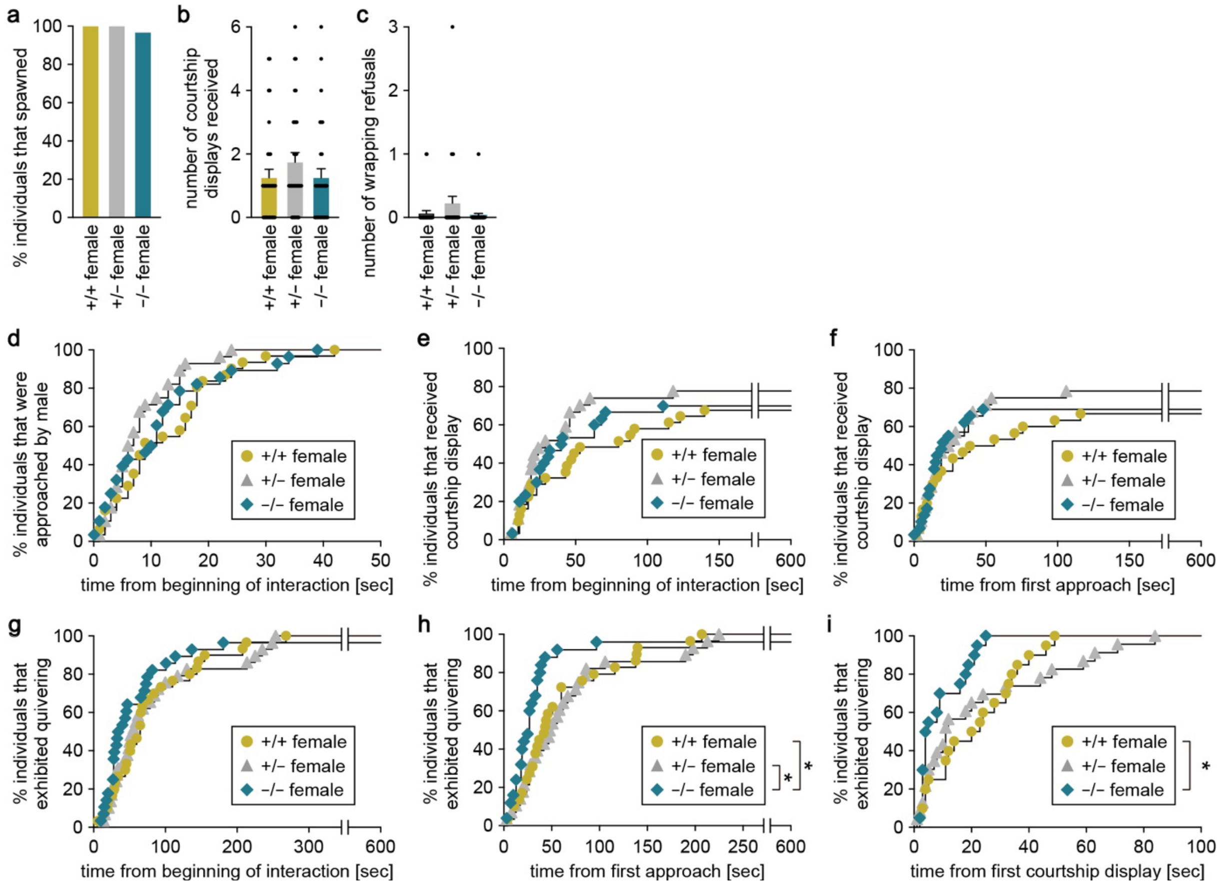


**Supplementary Fig. 3 *ptger4b*-deficient females exhibit increased sexual receptivity—Δ10 line.** *ptger4b*$^{+/+}$, *ptger4b*$^{+/-}$, and *ptger4b*$^{-/-}$ females of the Δ10 line were tested for mating behavior (n = 31 for each genotype). **a** Percentage of individuals that spawned during the test period (10 min). Number of courtship displays received (**b**) and wrapping refusals (**c**). Latency from the beginning of interaction to the first approach (**d**) and courtship display (**e**). **f** Latency from the first approach to the first courtship display. Latency from the beginning of interaction (**g**), first approach (**h**), and first courtship display (**i**) to quivering. Quantitative data were expressed as means with error bars representing standard error of the mean (**b**, **c**). Behavioral time-series data were expressed as Kaplan-Meier plots (**d**–**i**). Statistical differences were assessed by Fisher's exact test (**a**), Bonferroni's *post hoc* test (**b**, **c**), and Gehan-Breslow-Wilcoxon test with Bonferroni's correction (**d**–**i**). *$p < 0.05$.

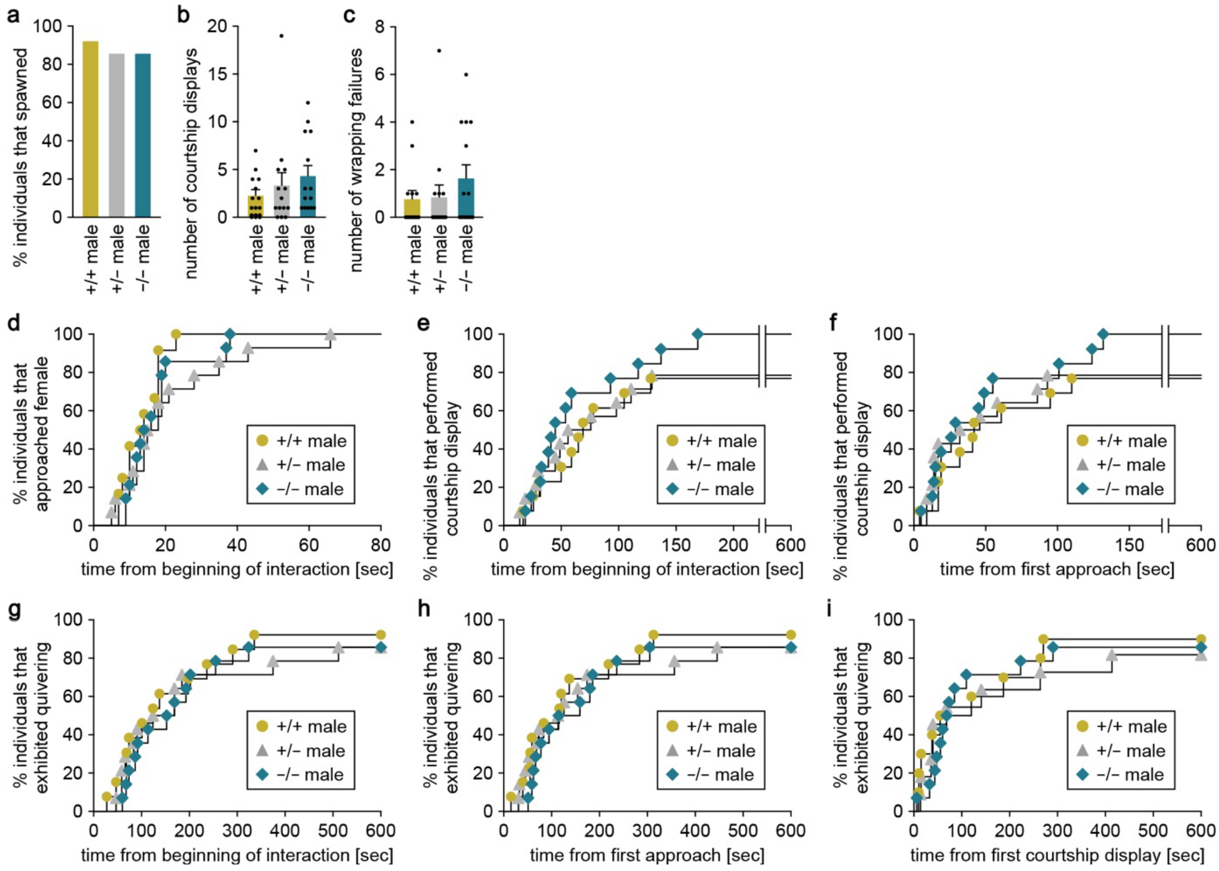


**Supplementary Fig. 4 Mating behavior of *ptger4b*-deficient males—Δ17 line.** *ptger4b*$^{+/+}$, *ptger4b*$^{+/-}$, and *ptger4b*$^{-/-}$ males of the Δ17 line were tested for mating behavior (n = 14 per genotype except for +/+, where n = 13). **a** Percentage of individuals that spawned during the test period (10 min). Number of courtship displays (**b**) and refused wrapping attempts (**c**). Latency from the beginning of interaction to the first approach (**d**) and courtship display (**e**). **f** Latency from the first approach to the first courtship display. Latency from the beginning of interaction (**g**), first approach (**h**), and first courtship display (**i**) to quivering. Quantitative data were expressed as means with error bars representing standard error of the mean (**b**, **c**). Behavioral time-series data were expressed as Kaplan-Meier plots (**d**–**i**). Statistical differences were assessed by Fisher's exact test (**a**), Bonferroni's *post hoc* test (**b**, **c**), and Gehan-Breslow-Wilcoxon test with Bonferroni's correction (**d**–**i**).

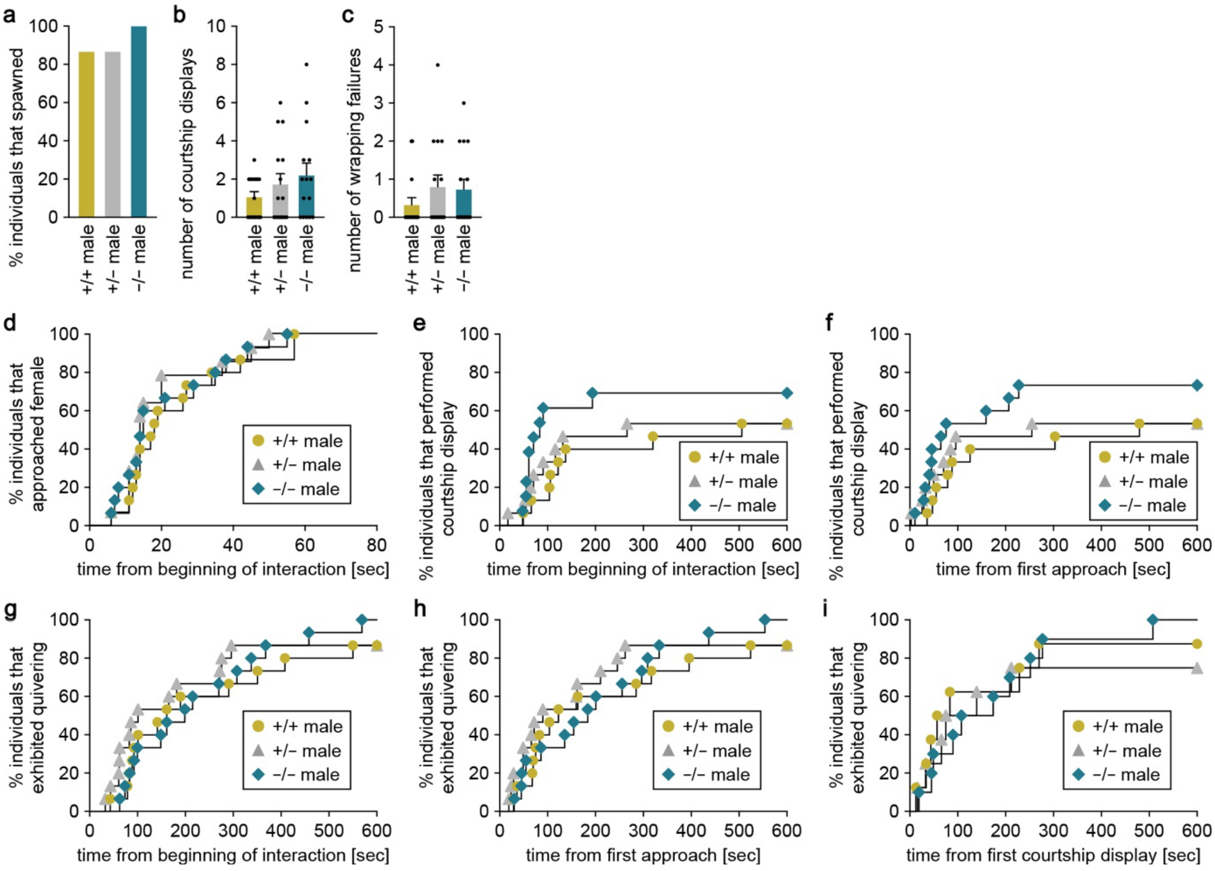


**Supplementary Fig. 5 Mating behavior of *ptger4b*-deficient males—Δ10 line.** *ptger4b*$^{+/+}$, *ptger4b*$^{+/-}$, and *ptger4b*$^{-/-}$ males of the Δ10 line were tested for mating behavior (n = 15 per genotype). **a** Percentage of individuals that spawned during the test period (10 min). Number of courtship displays (**b**) and refused wrapping attempts (**c**). Latency from the beginning of interaction to the first approach (**d**) and courtship display (**e**). **f** Latency from the first approach to the first courtship display. Latency from the beginning of interaction (**g**), first approach (**h**), and first courtship display (**i**) to quivering. Quantitative data were expressed as means with error bars representing standard error of the mean (**b**, **c**). Behavioral time-series data were expressed as Kaplan-Meier plots (**d**–**i**). Statistical differences were assessed by Fisher's exact test (**a**), Bonferroni's *post hoc* test (**b**, **c**), and Gehan-Breslow-Wilcoxon test with Bonferroni's correction (**d**–**i**).

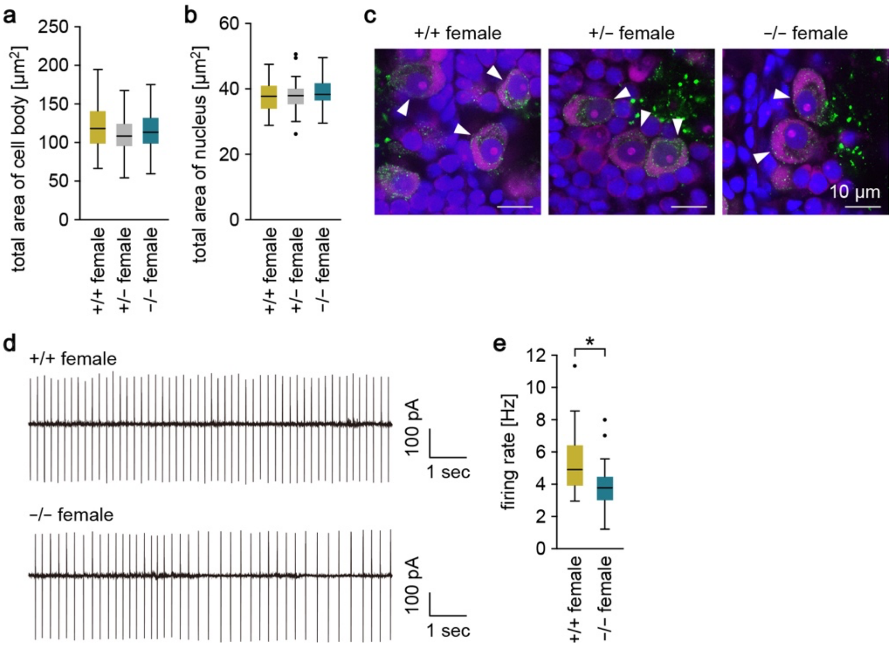


**Supplementary Fig. 6 *ptger4b* deficiency reduces firing activity in FeSP neurons—Δ10 line.** Cellular phenotypes of FeSP neurons in the PMm/PMg were analyzed in females of the Δ10 line. Cell body size (**a**) and nuclear size (**b**) of FeSP neurons in *ptger4b*$^{+/+}$, *ptger4b*$^{+/-}$, and *ptger4b*$^{-/-}$ females (n = 50 neurons from 5 individuals for each genotype). **c** Representative micrographs showing the morphology of FeSP neurons (arrowheads) in *ptger4b*$^{+/+}$, *ptger4b*$^{+/-}$, and *ptger4b*$^{-/-}$ females. Npba immunolabeling is shown in green; Nissl-stained cell bodies in magenta; and DAPI-stained nuclei in blue. Scale bars represent 10 μm. **d** Representative firing patterns of FeSP neurons in *ptger4b*$^{+/+}$ and *ptger4b*$^{-/-}$ females. **e** Firing frequencies of FeSP neurons in *ptger4b*$^{+/+}$ (n = 14 neurons from 7 individuals) and *ptger4b*$^{-/-}$ (n = 17 neurons from 7 individuals) females. Quantitative data were expressed as box-and-whisker plots. Statistical differences were assessed by Bonferroni's *post hoc* test (**a**, **b**) and unpaired *t*-test (**e**). *$p < 0.05$.

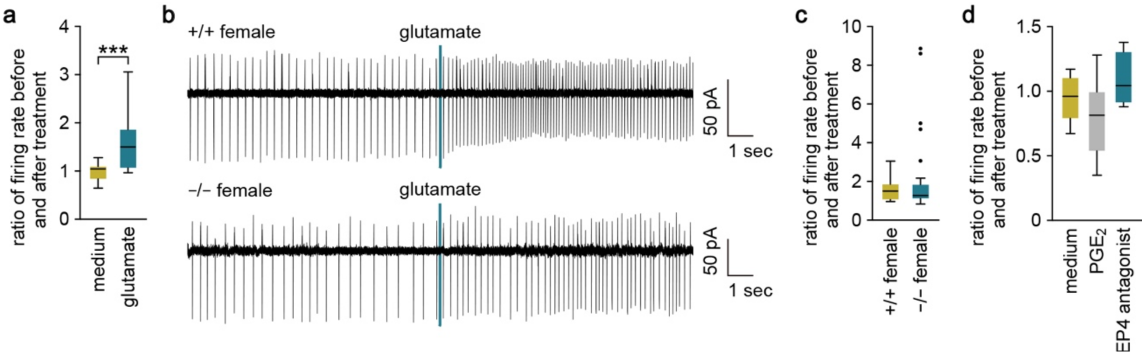


**Supplementary Fig. 7 *ptger4b* deficiency does not affect the response of FeSP neurons to glutamate.** The response of FeSP neurons to glutamatergic stimulation was investigated in females of the Δ17 line. **a** Ratio of firing rate of FeSP neurons before and after the application of medium only (n = 7 neurons from 4 individuals) or glutamate (n = 20 neurons from 8 individuals) in *ptger4b*$^{+/+}$ females. **b** Representative firing patterns of FeSP neurons in response to glutamate application in *ptger4b*$^{+/+}$ and *ptger4b*$^{-/-}$ females. **c** Ratio of firing rate of FeSP neurons before and after the application of glutamate in *ptger4b*$^{+/+}$ (n = 20 neurons from 8 individuals) and *ptger4b*$^{-/-}$ (n = 33 neurons from 14 individuals) females. **d** Ratio of the firing rates of FeSP neurons before and after the application of medium only (n = 10 from 8 individuals), $PGE_2$ (n = 8 from 8 individuals), or the EP4 antagonist GW 627368X (n = 4 from 3 individuals) in *ptger4b*$^{+/+}$ females. Quantitative data were expressed as box-and-whisker plots. Statistical differences were assessed by unpaired *t*-test with Welch's correction (**a**, **c**) and Dunnett's *post hoc* test (versus medium control) (**d**). ***$p < 0.001$.
