# Supplementary material for: Prostaglandin E2 receptor Ptger4b regulates female-specific peptidergic neurons and female sexual receptivity in medaka
Source: Commun Biol. 2022 Nov 10;5:1215. doi: 10.1038/s42003-022-04195-x (PMC9649691; doi:10.1038/s42003-022-04195-x)
Supplement: Supplementary file 3 — Description of Additional Supplementary Files [file 42003_2022_4195_MOESM3_ESM.pdf]

Description of Additional Supplementary Files

File name: Supplementary Data 1

Description: Summary of RNA sequencing.

File name: Supplementary Data 2

Description: The source data behind the graphs in the paper.
