# Supplementary material for: Prostaglandin E2 receptor Ptger4b regulates female-specific peptidergic neurons and female sexual receptivity in medaka
Source: Commun Biol. 2022 Nov 10;5:1215. doi: 10.1038/s42003-022-04195-x (PMC9649691; doi:10.1038/s42003-022-04195-x)
Supplement: Supplementary file 6 — Reporting Summary [file 42003_2022_4195_MOESM6_ESM.pdf]

nature portfolio

Corresponding author(s): Kataaki Okubo

Last updated by author(s): Oct 25, 2022

Reporting Summary

Nature Portfolio wishes to improve the reproducibility of the work that we publish. This form provides structure for consistency and transparency in reporting. For further information on Nature Portfolio policies, see our Editorial Policies and the Editorial Policy Checklist.

Statistics

For all statistical analyses, confirm that the following items are present in the figure legend, table legend, main text, or Methods section.

n/a	Confirmed	
☐	☒	The exact sample size (*n*) for each experimental group/condition, given as a discrete number and unit of measurement
☐	☒	A statement on whether measurements were taken from distinct samples or whether the same sample was measured repeatedly
☐	☒	The statistical test(s) used AND whether they are one- or two-sided<br>*Only common tests should be described solely by name; describe more complex techniques in the Methods section.*
☒	☐	A description of all covariates tested
☐	☒	A description of any assumptions or corrections, such as tests of normality and adjustment for multiple comparisons
☐	☒	A full description of the statistical parameters including central tendency (e.g. means) or other basic estimates (e.g. regression coefficient) AND variation (e.g. standard deviation) or associated estimates of uncertainty (e.g. confidence intervals)
☐	☒	For null hypothesis testing, the test statistic (e.g. *F*, *t*, *r*) with confidence intervals, effect sizes, degrees of freedom and *P* value noted<br>*Give P values as exact values whenever suitable.*
☒	☐	For Bayesian analysis, information on the choice of priors and Markov chain Monte Carlo settings
☒	☐	For hierarchical and complex designs, identification of the appropriate level for tests and full reporting of outcomes
☒	☐	Estimates of effect sizes (e.g. Cohen's *d*, Pearson's *r*), indicating how they were calculated

Our web collection on statistics for biologists contains articles on many of the points above.

Software and code

Policy information about availability of computer code

Data collection	TMHMM (ver. 2.0) (https://services.healthtech.dtu.dk); ClustalW (http://clustalw.ddbj.nig.ac.jp/index.php)
Data analysis	Adobe Photoshop (ver. 22); ImageJ (https://imagej.nih.gov/ij/); Clampfit (ver. 10.7); LAS X (ver. 3.7.4); GraphPad Prism (ver. 8)

For manuscripts utilizing custom algorithms or software that are central to the research but not yet described in published literature, software must be made available to editors and reviewers. We strongly encourage code deposition in a community repository (e.g. GitHub). See the Nature Portfolio guidelines for submitting code & software for further information.

Data

Policy information about availability of data

All manuscripts must include a data availability statement. This statement should provide the following information, where applicable:

- Accession codes, unique identifiers, or web links for publicly available datasets
- A description of any restrictions on data availability
- For clinical datasets or third party data, please ensure that the statement adheres to our policy

The RNA-seq data have been deposited in the DDBJ Sequenced Read Archive under the accession numbers DRR414603 to DRR414611. Source data for all graphs are provided in Supplementary Data 2. All other data supporting the findings of this study are available within the article and its supplementary information or can be provided from the corresponding author upon reasonable request.

Human research participants

Policy information about studies involving human research participants and Sex and Gender in Research.

Reporting on sex and gender	n/a
Population characteristics	n/a
Recruitment	n/a
Ethics oversight	n/a

Note that full information on the approval of the study protocol must also be provided in the manuscript.

Field-specific reporting

Please select the one below that is the best fit for your research. If you are not sure, read the appropriate sections before making your selection.

☒ Life sciences ☐ Behavioural & social sciences ☐ Ecological, evolutionary & environmental sciences

For a reference copy of the document with all sections, see nature.com/documents/nr-reporting-summary-flat.pdf

Life sciences study design

All studies must disclose on these points even when the disclosure is negative.

Sample size	Power analysis was not performed prior to beginning the study due to the lack of existing data on medaka FeSP neurons. The sample size was estimated on the basis of our previous studies which provided information on inter-individual variation in neural gene expression levels in medaka (e.g., Hiraki-Kajiyama et al., 2019, eLife, 8:e39495; Yamashita et al., 2020, eLife, e59470; Nishiike et al., 2021, Curr Biol, 31:1699-1710).
Data exclusions	Statistical outliers in the behavioral data were determined with a ROUT test, using a false-positive rate (Q) of 0.1% and removed from the data sets.
Replication	Since the analysis on different individuals yielded similar results, we believe that the results are reproducible.
Randomization	Animals were randomly assigned to experimental groups.
Blinding	Blinding was not performed because most of the data collection and analysis was done by automated software.

Reporting for specific materials, systems and methods

We require information from authors about some types of materials, experimental systems and methods used in many studies. Here, indicate whether each material, system or method listed is relevant to your study. If you are not sure if a list item applies to your research, read the appropriate section before selecting a response.

Materials & experimental systems

n/a	Involved in the study	
☐	☒	Antibodies
☐	☒	Eukaryotic cell lines
☒	☐	Palaeontology and archaeology
☐	☒	Animals and other organisms
☒	☐	Clinical data
☒	☐	Dual use research of concern

Methods

n/a	Involved in the study	
☒	☐	ChIP-seq
☒	☐	Flow cytometry
☒	☐	MRI-based neuroimaging

Antibodies

Antibodies used	Horseradish peroxidase-conjugated anti-fluorescein antibody (RRID: AB_2737388; PerkinElmer); alkaline phosphatase-conjugated anti-DIG antibody (RRID: AB_514497; Roche Diagnostics); Alexa Fluor 488-conjugated goat anti-rabbit IgG (RRID: AB_2534114; Thermo Fisher Scientific); Alexa Fluor 555-conjugated goat anti-rabbit IgG (RRID: AB_2535849; Thermo Fisher Scientific); rabbit anti-Npb polyclonal antibody (RRID: AB_2810229)

Validation	The anti-fluorescein, DIG, and rabbit IgG antibodies are all known to have high specificity and are commonly used. The anti-Npb antibody has been shown to recognize medaka Npba with high specificity (Hiraki-Kajiyama et al., 2019, eLife, 8:e39495).

Eukaryotic cell lines

Policy information about cell lines and Sex and Gender in Research

Cell line source(s)	CHO cells: Riken BRC Cell Bank
Authentication	Cells were authenticated by the supplier (Riken BRC Cell Bank).
Mycoplasma contamination	Cells were confirmed to be mycoplasma free by the supplier (Riken BRC Cell Bank).
Commonly misidentified lines (See ICLAC register)	n/a

Animals and other research organisms

Policy information about studies involving animals; ARRIVE guidelines recommended for reporting animal research, and Sex and Gender in Research

Laboratory animals	Species: Medaka (Oryzias latipes)<br>Strain: Wild-type d-rR strain, npba-GFP transgenic line, and ptger4 knockout lines (Δ17 and Δ10 lines) were used.<br>Sex: Fish of both sexes were used in all analyses except for those of FeSP neurons, which occur only in females.<br>Age: Spawning adult fish (aged 3–5 months) were used in all analyses.
Wild animals	n/a
Reporting on sex	Because this study focused on neurons occurring only in females, many of the analyses were performed only in females.
Field-collected samples	n/a
Ethics oversight	n/a

Note that full information on the approval of the study protocol must also be provided in the manuscript.
